# Effects of heat stress on oxidative balance and sperm quality in dogs

**DOI:** 10.3389/fvets.2024.1445058

**Published:** 2024-09-26

**Authors:** Matteo Burgio, Lucrezia Forte, Andrea Prete, Aristide Maggiolino, Pasquale De Palo, Giulio Guido Aiudi, Annalisa Rizzo, Alice Carbonari, Giovanni Michele Lacalandra, Vincenzo Cicirelli

**Affiliations:** Department of Veterinary Medicine, University of Bari A. Moro, Valenzano, Italy

**Keywords:** heat stress, sperm quality dog, ROS, biochemical profile, antioxidant potential

## Abstract

**Introduction:**

Heat stress negatively affects both animal reproductive performance and their overall wellbeing and welfare. When temperatures soar, the body responds to maintain balance, resulting in heat stress. This triggers various responses, including the buildup of reactive oxygen species (ROS), which can harm sperm vitality through lipid peroxidation. Oxidative damage can result in sperm dysfunction. This study aimed to evaluate the effects of environmental heat stress on canine quantitative and qualitative ejaculate parameters.

**Methods:**

Thirty-six male crossbred dogs were involved in the trial. This study was performed in 2022, precisely from May to August. Dogs were subdivided in two groups, one heat stressed (HS) and one in thermoneutrality (TN). Thermo Hygrometric Index (THI) was hourly measured and ranged from 60 to 71 in TN dogs and from 60 to 83 for HS dogs. Semen and blood samples were collected at 30-day intervals, starting from May (0 days), and then at 30 days, 60 days, and 90 days and analysis for evaluating biochemical profile, semen oxidative status, and semen quality were performed.

**Results:**

In HS dogs, serum total protein, albumin, and urea concentrations showed a significant decrease after 60 days (*P* < 0.01), with values lower than those observed in TN dogs (*P* < 0.01). Both catalase and glutathione peroxidase concentrations were reduced after 60 days in HS dogs, showing lower levels than the TN group (*P* < 0.01 and *P* < 0.05, respectively). Antioxidant potential increased over time in HS dogs, reaching higher values at 60 days (*P* < 0.05) and 90 days (*P* < 0.01). On the other hand, ROS in the sperm of HS animals rose by day 90, surpassing the values recorded at previous time points and in TN dogs (*P* < 0.01). Semen concentration (*P* > 0.01) and total sperm count (*P* < 0.05) declined after 30 days in the HS group and remained lower than the TN group throughout the trial.

**Discussion:**

The study demonstrates that heat stress negatively affects the oxidative status and sperm quality of male dogs, reducing reproductive performance. However, further research is needed due to the lack of complete breed homogeneity in the study groups.

## 1 Introduction

It is now widely recognized within the scientific community that our planet is experiencing global climate change (1–3). Since the industrial revolution, human activities have contributed to a rise in global temperatures (2). This increase has exceeded 1°C since 1950, with the last 7 years being the hottest on record (4, 5). In response to the growing challenge posed by climate change, there has been increased focus on the relationships among heat stress, behavior, coat and skin characteristics, thermoregulatory ability, oxidative stress, and fertility (6, 71). For dogs, a Thermo Hygrometric Index (THI) value exceeding 72–74 is generally considered a critical threshold beyond which heat stress begins. This value serves as a general guideline and can vary based on factors such as breed, age, health, and the physical condition of the animal. Brachycephalic breeds (such as Bulldogs and Pugs) may be more sensitive to heat compared to other breeds (46, 69). In regions affected by climate change, heat stress is the primary factor responsible for declines in fertility and productivity (1, 70). The current harshening of heat stress scenarios negatively impacts reproduction (7). Heat stress promotes the formation of reactive oxygen species (ROS) (8), which primarily target the polyunsaturated fatty acids found in the membrane phospholipids of spermatozoa. The modification of these fatty acids leads to the disruption of cell structure and function. Specifically, heat stress-induced ROS generation can result in decreased sperm concentration, average pathway velocity, straight-line velocity, curvilinear velocity, amplitude of lateral head displacement, straightness, and linearity. This oxidative stress not only causes cellular damage but also initiates the process of apoptosis (9). Testes are particularly susceptible to hyperthermia, as the pathway from spermatogonia to spermatozoa involves numerous cellular divisions, including the loss of cellular machinery and high DNA condensation, making this process highly vulnerable to external threats such as elevated temperatures (10–12). Ideally, testicular temperature should be maintained 2 to 8°C below body temperature in various species [boar: (13); bull: (14); ram: (15); stallion: (16); dog: (17)]. To protect against environmental heat stress, the testes utilize a sophisticated thermal regulation mechanism. The scrotal skin, which is thin, with minimal adipose tissue and hair, and a well-developed blood and lymphatic system, plays a crucial role in maintaining lower testicular temperatures. These characteristics facilitate heat dissipation through radiation and evaporation (18). Additionally, the pampiniform plexus acts as a heat exchange mechanism that allow cooling of the arterial blood entering the testis. The cremaster muscle and dartos tunic work together to retract and relax the testes relative to the abdomen, further aiding in temperature regulation (19, 20). However, when temperatures rise beyond the regulatory capacity of these mechanisms, it can lead to a range of reproductive issues. In dogs, elevated temperatures are associated with reduced libido, impaired spermatogenesis, lower sperm concentration, poor sperm quality, decreased testicular weight, and a temporary period of partial or total infertility (14, 16, 21–23). Sperm parameters, including motility and vigor, may be negatively impacted, while morphological defects such as acrosome degradation, proximal cytoplasmic droplets, bent tails at the head, small heads, and isolated heads become more prevalent, as observed in bulls (24). Despite these significant findings, there is limited research (17) on the effects of heat stress on the health and semen quality of dogs. This study, therefore, aims to assess the impact of high environmental temperatures on clinical and biochemical parameters, oxidative status, and sperm quality in dogs. However, it is important to note that the study faces limitations due to challenges in standardizing the groups, particularly in terms of the dogs' breeds and environmental conditions. Since the dogs involved are privately owned, this introduces a small but unpredictable variable, as their living conditions, while similar, cannot be perfectly controlled.

## 2 Materials and methods

### 2.1 Ethics

This study was performed in accordance with the ethical guidelines of the Animal Welfare Committee. Institutional Review Board approval of the study was obtained with approval number (656/18). Informed owner consent was obtained for all dogs.

### 2.2 Animals and experimental design

**Figure 1:**
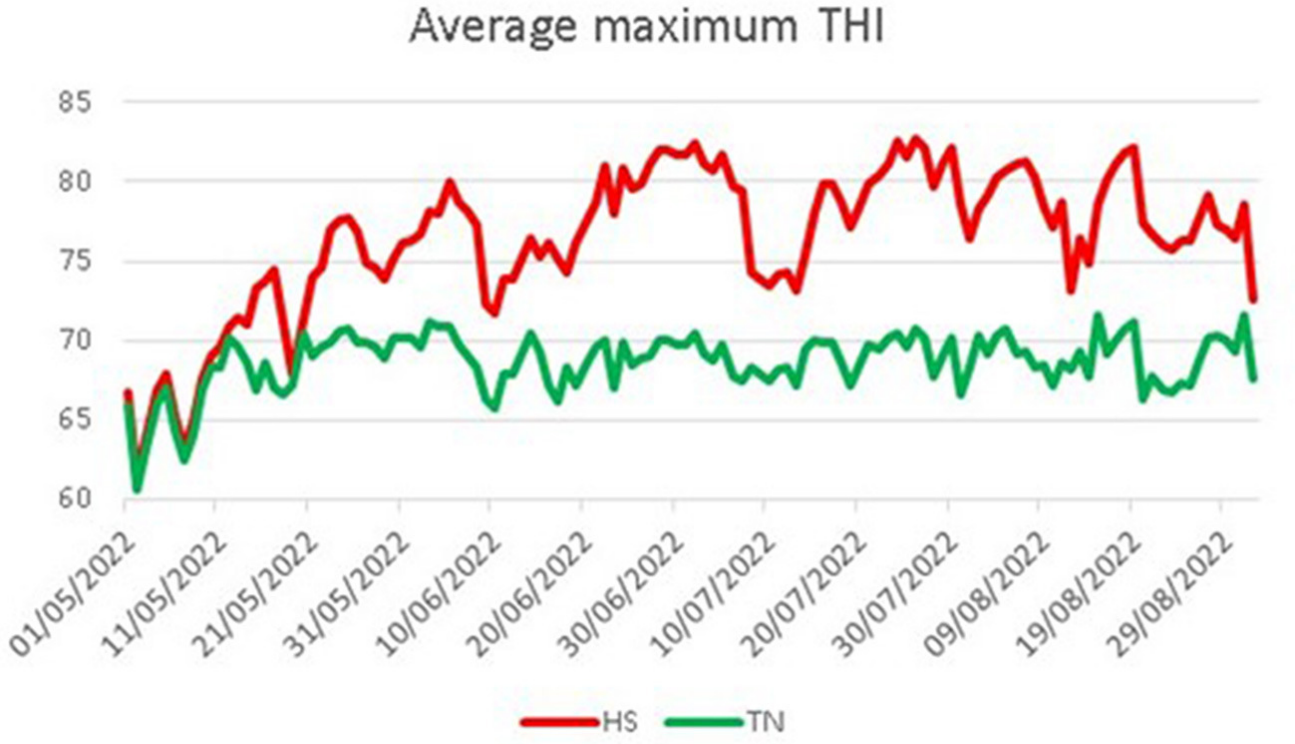
Average maximum THI registered for the two experimental groups. HS, heat stress; TN, thermoneutrality.

The study was performed from May to August 2022, in the south of Italy. A total of 36 male dogs, body condition score of 3 (±0.5), were involved in the trial. All dogs were mixed breeds, owned; 18 were consistently kept indoors with functioning air conditioning systems (thermoneutrality group, TN), while 18 were consistently kept outdoors (heat stress group, HS) (Table 1 reported all animals characteristics). All animals were individually housed. Groups were balanced for coat length and color. All dogs were clinically examined to ensure their health status 30 days before the beginning of the trial. Each dog has submitted for a clinical examination and blood analyses. An ultrasound exam of the re-productive tract was also performed, and it was verified that all dogs reacted positively to sperm collection by digital manipulation. Exclusion criteria included obesity, white or long hair, and use of medications within the previous 30 days. All owners were provided with a temperature and humidity monitoring system (Tinytag from Data Loggers, Gemini Data Loggers Ltd, West Sussex, United Kingdom), placed within the indoor space for the TN group dogs and in the outdoor area for the HS group dogs. The data loggers were set to record temperature and humidity on an hourly basis. During each control time for semen and blood sampling, the data logger was brought to the Obstetric, Gynecological, and Andrological Clinic of the Veterinary Medicine Department of the “Aldo Moro” University of Bari (Italy), and the data were downloaded to calculate the Temperature Humidity Index (THI). This dataset was employed to compute the hourly THI utilizing the formula outlined by Maggiolino et al. (25):


$$\begin{array}{cc} \text{THI } & =\;\left(1.8\;\times \text{ AT }+\;32\right)\;-\;\left(0.55\;-\;0.0055\;\times \text{ RH}\right)\\ \; & \times \;\left[\left(1.8\;\times \text{ AT }+\;32\right)\;-\;58\right] \end{array}$$

where AT is the environmental temperature expressed in degrees Celsius, so that the term (1.8 × AT + 32) represents the conversion of temperature data in degrees Fahrenheit, and RH is the relative humidity as a fraction of unit. Average maximum THI values registered for each experimental group were reported in Figure 1. All dogs were fed a standardized feed starting 30 days before the beginning of the trial [10 g/kg of body weight (BW) daily, composition reported in Table 2] two times a day.

**Table 1:** Sample characteristics by group (TN vs. HS).

**Variable**	**Group**	
	**TN (*****n*** = **18)**	**HS (*****n*** = **18)**
Age (years)	3 ± 0.2	3 ± 0.8
Weight (kg)	20 ± 0.5	23 ± 0.6

TN, thermoneutrality; HS, heat stress.

**Table 2:** Composition of diet commercial feed.

**Item**	**On 100 g of product**
Moisture	10 g
Raw protein	28 g
Fat	15 g
Raw fibers	3 g
Ashes	9 g
Vitamin A	1,500 IU
Vitamin D	100 IU
Vitamin E acetate (alpha-tocopherol 91%)	12.5 mg
Vitamin B2	2.6 mg
Vitamin B6 (pyridoxine hydrochloride)	0.5 mg
Vitamin B1 (thiamine mononitrate)	0.6 mg
Choline chloride	75 mg
Iodine (anhydrous calcium iodate)	0.075 mg
D-panthotenic acid	1 mg
Vitamin H (Biotin D)	0.05 mg
Calcium	0.5 g
Vitamin K3 (menadione)	0.125 mg
Vitamin PP (nicotine acid)	2.5 mg
Vitamin B12	0.0035 mg
Folic acid	0.1 mg
Cobalt (basic cobalt carbonate)	0.015 mg
Iron (ferrous carbonate)	2 mg
Manganese (manganous oxide)	4 mg
Copper (Copper sulfate, pentahydrate)	1 mg
Selenium (sodium selenite)	0.01 mg
Zinc (zinc oxide)	3 mg

**Figure 2:**
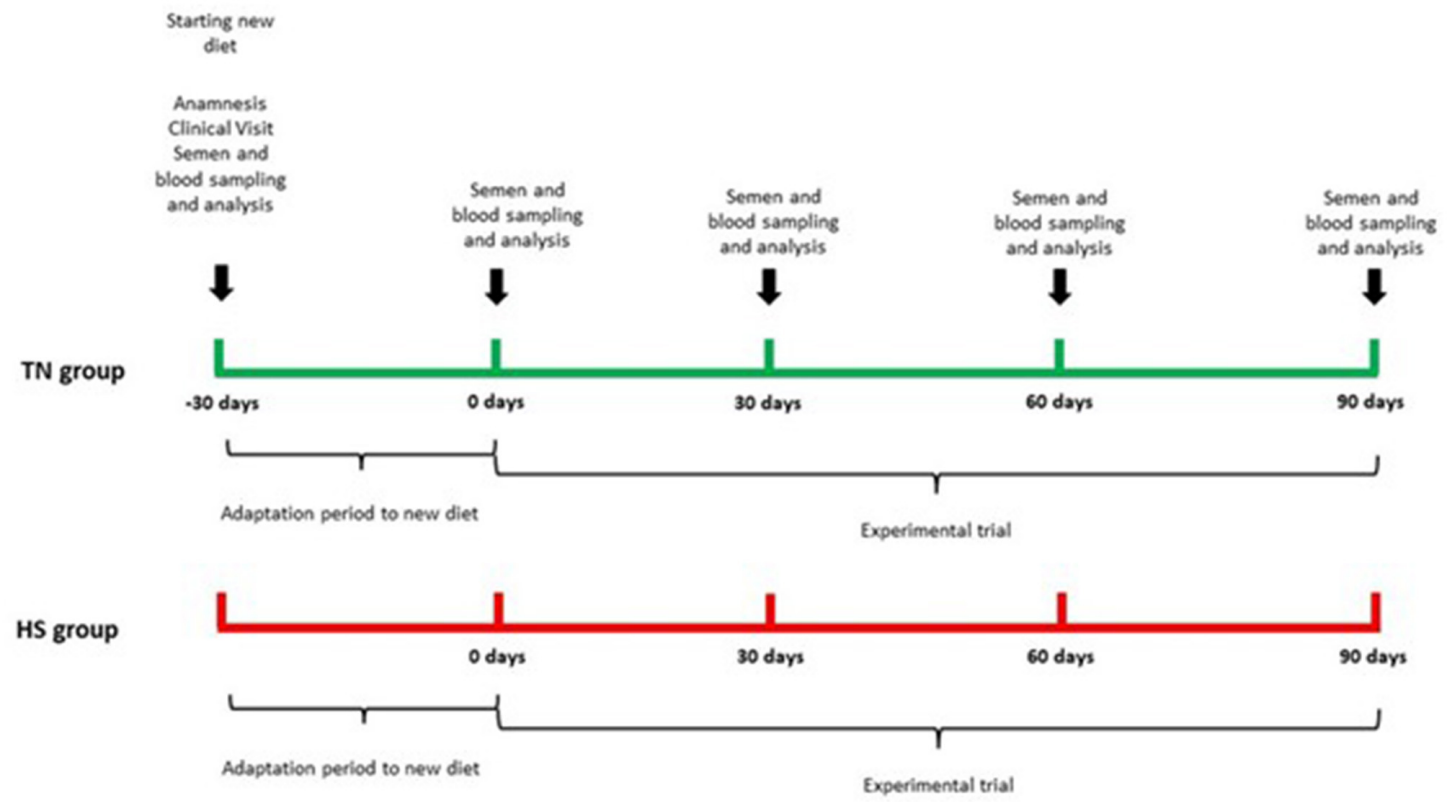
Experimental design.

Semen and blood samples were collected on day 0, 30, 60, and 90 (Figure 2).

The trial lasted 120 days, after 30 days of adaptation to new diet, there was our starting time point (T0) and all sampling procedures and analysis were performed every 30 days (T30, T60, T90) until the end of the trial. The experimental design is reported in Figure 2.

### 2.3 Blood samples and analysis

Blood was collected aseptically via puncture of the cephalic vein using 22-gauge needles, employing a negative pressure 4 ml tube system for serum (without anticoagulant) and plasma (with 15 USP U/ml of heparin) (Becton, Dickinson Canada Inc, Vacutainer 1, Oakville, Canada). Tubes intended for plasma were promptly centrifuged (1,500 × g for 10 min), while tubes for serum were allowed to clot at a refrigerated temperature for 10 min before being centrifuged (1,500 × g for 10 min). All plasma and serum aliquots were stored at −80°C until analysis (26). All blood samples were taken in the morning by 10 am with the animals fasting for at least 8 h.

#### 2.3.1 Biochemical profile

Clinical biochemistry parameters were obtained from the serum samples using an automated biochemistry analyzer (CS-300B; Dirui, Changchun, China) as described by De Palo et al. (27). The following parameters were analyzed: alanine aminotransferase (ALT), aspartate aminotransferase (AST), creatine phosphokinase (CPK), lactate dehydrogenase (LDH), alkaline phosphatase (ALP), glucose (Glu), blood urea nitrogen (BUN), creatinine (Crea), total serum protein (TP), albumin (Alb), cholesterol (Chol), triglycerides (Trig), non-esterified fatty acids (NEFA), calcium (Ca), phosphate (P), magnesium (Mg), chloride (Cl), (Gesan Production Kit, Campobello di Mazara, Trapani, Italy). Globulins (Glob) and albumin/globulin ratio (Alb/Glob) were calculated starting from total protein and albumin parameters. Cortisol was determined using a commercial radioimmunoassay kit (Coat-a-count Cortisol, Siemens Medical Solution Diagnostics, Los Angeles, CA), according to the manufacturer's protocol (28).

#### 2.3.2 Oxidation parameters and enzymes activity

Plasma samples were used for oxidation parameters and antioxidant enzyme activities assays. Thiobarbituric acid reactive substances (TBARs) were determined spectrophotometrically, by adding 100 ml of plasma to a 3.7 μl/ml thiobarbituric acid solution. Plasma reactive carbonyl derivative (RCD) levels were determined according to Faure and Lafond (29) using the carbonyl reagent DNPH as described by Forte et al. (30). The absorbance was measured at 380 nm. Hydroperoxides (Hy) were determined spectrophotometrically by an iodometric method as described by Maggiolino et al. (31). Protein carbonyls (PC) levels were determined spectrophotometrically as reported by Salzano et al. (72). The superoxide dismutase (SOD) (EC 1.15.1.1) activity was examined according to Misra (32), and the enzymatic activity was based on the 50% inhibition rate of epinephrine autooxidation at 480 nm. The catalase (CAT) (EC 1.11.1.6) activity was assayed by the method of Clairborne (33). The glutathione peroxidase (GPx) (EC1.11.1.9.) activity was measured according to Dinardo et al. (34).

#### 2.3.3 ROS and BAP determination

The ROS and BAP (biological antioxidant potential) serum concentrations were obtained by the means of a photometric analytical system (FREE Carpe Diem^®^, Diacron International srl, Grosseto, Italy) (35). The d-ROMs (reactive oxygen metabolites) test determines the concentration of reactive oxygen metabolites (ROMs) in a biological sample, particularly hydroperoxides, deriving from the oxidative attack of many bio-chemical substrates (glycids, lipids, amino acids, proteins, nucleotides, etc.) (36). They were measured using a free radical elective evaluator (FREE Carpe Diem; Diacron International) that included a spectrophotometric device reader, and measurement kits (d-ROMs test, Wismerll Co. Ltd., Tokyo, Japan) were optimized to the FREE Carpe Diem^®^ System according to the manufacturer's protocol. Briefly, a 20 μl plasma sample and 1 mL buffered solution (R2 kit reagent, pH 4.8) were gently mixed in a cuvette, and 20 μl of chromogenic substrate (R1 kit reagent) was then added to the cuvette (35, 36). After mixing well, the cuvette was immediately incubated in the thermostatic block of the analyzer for 5 min at 37°C, and absorbance at 505 nm was recorded. The results were expressed in arbitrary units, the Carratelli Units (U/CARR), one unit corresponds to 0.8 mg/L of hydrogen peroxide. The BAP was measured using a free radical elective evaluator (FREE Carpe Diem^®^) that included a spectrophotometric device reader, and measurement kits (BAP test) (Wismerll Co. Ltd.) were optimized to the FREE Carpe Diem^®^ System according to the manufacturer's protocol. Briefly, 50 μl of chromogenic substrate (R2 kit reagent) and 1 ml of reactive solution (R1 kit reagent) were gently mixed in a cuvette, and absorbance at 505 nm was recorded. A 10 μl plasma sample was then immediately added to the cuvette. After mixing, the cuvette was immediately incubated in the thermostatic block of the analyzer for 5 min at 37°C, and absorbance at 505 nm was recorded. The results were expressed as mmol/L of reduced ferric ions (35, 36).

### 2.4 Semen collection and computer assisted sperm analysis

Dog's semen was collected into a sterile container by manual stimulation, while the dogs sniffed swabs of bitches in estrous. The semen collection was divided in three Falcon tubes, one for each part of the ejaculate: urethral, spermatic, and prostatic (37). The ejaculation analysis was performed as described by Alonge et al. (38). Only the spermatic part was analyzed by the Computer Assisted Sperm Analyzer (39) (CASA, IVOS-Sperm CASA system, Version 12.3, Hamilton Thorne, MA, USA). The CASA software (IVOS 12.3 version) was set up for canine semen specific parameters (Table 3). According to the manufacturer's instructions, for each analysis, a 3 μl drop from each sperm sample was diluted 5 times in Tris-Fructose extender and put on a Leja slide (four chambers) of 20 μm (Leja Products B.V. Nieuw Vennep, The Netherlands). The Leja slide was positioned in the dedicated chamber of the microscope, allowing it to settle for a few seconds before analysis. The CASA scanned five random non-consecutive microscopic fields. The parameters evaluated were total number of counted cells (TSC); semen concentration; total motility, and percentage of motile spermatozoa (progressive motility). Velocity average pathway (VAP) was elaborated by the software as average velocity of smoothed cell path, expressed in m/s. Then, the overall sperm population was divided into four groups, based on the velocity, according to low VAP cut-off (LVV) and medium VAP cut-off (MVV). Thus, sperms were classified as follows: rapid spermatozoa, with VAP > MVV; medium spermatozoa, with LVV < VAP < MVV; slow spermatozoa, with VAP < LVV; and static spermatozoa, represented by the fraction of those cells not moving during the analysis.

**Table 3:** IVOS 12,3 software settings for dog semen parameters.

**Parameters**	**Cut-off value**
Frames per second (Fps)	30
Frequency	60 Hz
Temperature of analysis	37°C
Minimum contrast	75
Minimum cell size	4 pixels
Progressive cell cut-off	100 μm/s; 75% STR
Low VAP cut-off	9 μm/s
Low VSL cut-off	20 μm/s

### 2.5 Statistical analysis

Each animal represented an experimental unit. All data sets were tested for normal distribution (Shapiro-Wilk) and variance homogeneity (Bartlett test). All parameters were subjected to analysis of variance (ANOVA) according to the General Linear Model (GLM) procedure as reported the following model:


$$\begin{array}{l} \text{yijk}=\text{μ}+\text{αi}+\text{Gj}+\text{Tk}+\left(\text{G}\times \text{T}\right)\text{jk}+\text{ εijkl}, \end{array}$$

where yijk represents all blood variables; μ is the overall mean; αi is the constant of the individual dog random effect (i = 1,…, 36); G represents the effect of the jth group (j = 1, 2), T was the effect of the kth time (k = 1, …, 4), G × T represents the binary interaction between the jth group and the kth time (1,…, 8). Significance was set at *P* < 0.05, and the results were expressed as means and mean standard error. All the analysis were per-formed using SAS software (40).

## 3 Results

The results concerning the clinical biochemical profile of the dogs are reported in Tables 4, 5. Total protein, albumin, urea, glycemia, ALT, AST and ALP were affected by heat stress, time exposure and their binary interaction (*P* < 0.01). Serum total protein, albumin and urea concentrations decreased after 60 days in HS dogs (*P* < 0.01), showing lower values than TN (*P* < 0.01). Glycemia decreased as early as 30 days in HS dogs (*P* < 0.01), maintaining lower values than TN dogs from 30 to 90 days of trial (*P* < 0.01). The ALT, AST, and ALP concentration, instead, increased in HS dogs after 30 days of trial (*P* < 0.01), showing higher values than TN from 30 to 90 days of trial (*P* < 0.01). Cortisol values and oxidative profile results are reported in Table 6. Cortisol, TBARs, SOD, CAT and GSPx plasma concentration were affected by heat stress, time exposure and their binary interaction (*P* < 0.01). Cortisol plasma concentration increased constantly until 60 days of trial in HS dogs (*P* < 0.01) and then remained constant, showing higher values than TN from 30 to 90 days (*P* < 0.01). TBARs concentration increased at 30 days in HS dogs (*P* < 0.01), showing higher values than TN group from 30 to 90 days (*P* < 0.01). The SOD concentration decreased after 30 days of trial in HS dogs, with lower values than TN until the end of the trial (*P* < 0.01). Differently, CAT and GSPx concentration decreased after 60 days in HS dogs, with lower values than TN group (respectively *P* < 0.01 and P < 0.05). Bap values increased during time in HS animals, recording at 60 (*P* < 0.05) and 90 (*P* < 0.01) days higher values than previous experimental times. Moreover, at 60 (*P* < 0.05) and 90 (*P* < 0.01) days, these values were lower in HS dogs compared to TN ones. Differently, ROS values increased in spermatozoa of HS animals at 90 days compared to previous days and to TN animals (*P* < 0.01). Tables 7, 8 showed semen parameters. Semen concentration (*P* > 0.01) and TSN (*P* < 0.05) decreased after 30 days in HS dogs, and values remained lower than TN group until the end of the trial. Differently, VAP values did not show to be affected by time (*P* > 0.05), but HS dogs registered lower values than TN ones at 30 (*P* < 0.05), 60 and 90 days (*P* < 01). VCL values decreased at 90 days compared to 30 days in TN group (*P* < 0.01) and at 30 days in the HS group (*P* < 0.05) and then remains constant, with lower values in HS than TN dogs only at 30 days (*P* < 0.05). STR values of HS animals at 90 days were lower than what observed at 0, 30 (*P* < 0.01) and 60 days (*P* < 0.05) in same dogs and then TN dogs at same time (*P* < 0.01). The LIN values of HS dogs were lower at 90 days than 0 days of same dogs and then TN dogs at same time (*P* < 0.05). Total motility de-creased in HS dogs after 90 days compared to 0 and 30 days (*P* < 0.01). These animals showed lower values at 60 and 90 days compared to TN dogs (*P* < 0.01). Also, progressive motility decreased in HS dogs during time, with lower values at 60 days (*P* < 0.05) and 90 days (*P* < 0.01) compared to 0 and 30 days. Moreover, from 60 to 90 days HS animals showed lower progressive motility than TN ones (*P* < 0.01). Rapid movements observed in HS animals decreased at 30, 60 (*P* < 0.05) and 90 days (*P* < 0.01) compared to 0 days, with lower values from 30 to 90 days than what registered in TN animals (*P* < 0.01). Contrarily, slow movements increased in HS animals just after 30 days (*P* < 0.01) and remained constant with higher values than TN dogs from 30 to 90 days (*P* < 0.01). Static movements, instead, were constantly higher in HS animals from 30 to 90 days of the trial (*P* < 0.05).

**Table 4:** Total protein, albumin, urea, uric acid, creatinine, bilirubin, triglyceride, glycemia, alanine amino transferase (ALT), aspartate amino transferase (AST), alkaline phosphatase (ALP), and cholesterol serum concentration in dogs in thermoneutrality (TN) and heat stress (HS) condition for 90 days.

**Parameter**	**Group**	**Time (days)**				**SEM**	***p*** **-value**			**Reference value^a^**
		**0**	**30**	**60**	**90**		**G**	**T**	**G** × **T**	
Total protein (g/dL)	TN	6.41	6.61	7.33^X^	6.44^X^	0.32	< 0.001	< 0.001	< 0.001	5.4–7.1
	HS	6.43^A^	6.05	5.47^B, Y^	5.21^B, Y^					
Albumin (g/dL)	TN	3.21	2.91	3.28^X^	2.88^X^	0.10	< 0.001	< 0.001	< 0.001	2.6–3.3
	HS	2.99^A^	2.68^A^	2.32^B, Y^	2.28^B, Y^					
Urea (mmol/L)	TN	58.22	58.92	54.74^X^	61.08^X^	4.89	0.009	< 0.001	< 0.001	21–60
	HS	56.17^A^	55.95^A^	42.94^B, Y^	39.37^B, Y^					
Uric acid (mg/dL)	TN	0.79	0.87	0.98	0.96	0.19	0.6361	0.2022	0.5341	0.0–2.0
	HS	0.62	0.83	0.97	0.93					
Creatinine (mg/dL)	TN	1.28	1.26	1.18	1.08	0.10	0.0474	0.4732	0.5294	0.5–1.5
	HS	1.25	1.32	1.17	1.13					
Bilirubine (mg/dL)	TN	0.22	0.32	0.27	0.28	0.13	0.4562	0.1354	0.4262	0.1–0.5
	HS	0.19	0.28	0.24	0.25					
Triglyceride (mg/dL)	TN	38.63	38.65	35.65	37.33	3.10	0.3374	0.1792	0.5424	30.1–38.1
	HS	36.95	32.35	32.5	31.28					
Glycemia (mg/dL)	TN	97.03	104.26^X^	99.67^X^	101.74^X^	5.10	< 0.001	< 0.001	< 0.001	65–118
	HS	99.41^A^	76.71^B, Y^	67.03^B, Y^	68.69^B, Y^					
ALT (IU/L)	TN	31.78	38.47^X^	43.24^X^	36.66^X^	1.75	0.0021	0.0002	< 0.001	21–102
	HS	34.69^A^	53.23^B, Y^	65.48^B, Y^	65.25^B, Y^					
AST (IU/L)	TN	24.74	25.44^X^	26.93^X^	27.95^X^	2.21	< 0.001	< 0.001	< 0.001	0.0–66.0
	HS	21.73^A^	43.84^B, Y^	41.5^B, Y^	43.71^B, Y^					
ALP (IU/L)	TN	22.58	20.11^X^	23.49^X^	16.59^X^	1.16	< 0.001	< 0.001	< 0.001	20–156
	HS	23.36^A^	33.76^B, Y^	35.99^B, Y^	38.79^B, Y^					
Cholesterol (mg/dL)	TN	181.6	184.97	201.9	192.41	18.65	0.7864	0.3741	0.7473	135–270
	HS	179.4	182.88	187.83	186.87					

SEM, standard error of the means; G, Group; T, Time; ALT, alanine aminotransferase; AST, aspartate aminotransferase; ALP, alkaline phosphatase.

^a^Kaneko (42).

Different letters on the same parameter of the same group show statistical differences: A, B = *P* < 0.01. Different letters at the same time for the same parameter show statistical differences: X, Y = *P* < 0.01.

**Table 5:** Chlorine, sodium, potassium, magnesium, phosphorus, and calcium serum concentration in dogs in thermoneutrality (TN) and heat stress (HS) condition for 90 days.

**Parameter**	**Group**	**Time (days)**				**SEM**	***p*** **-value**			**Reference value^a^**
		**0**	**30**	**60**	**90**		**G**	**T**	**G** × **T**	
Chlorine (mEq/L)	TN	112.35	113.15	114.93^X^	109.7^X^	2.85	< 0.001	< 0.001	< 0.001	105.0–115.0
	HS	114.67	111.05	98.83^B, Y^	91.43^B, Y^					
Sodium (mmol/L)	TN	143.54	148.61	144.81	141.34	3.03	0.4211	0.9354	0.1991	141.0–152.0
	HS	143.07	148.77	144.55	144.43					
Potassium (mg/dL)	TN	4.42	4.41	4.53	4.47	0.13	0.2411	0.6222	0.6591	4.4–5.3
	HS	4.1	4.09	4.12	4.15					
Magnesium (mg/dL)	TN	2.06	1.85	2.22	2.22	0.43	0.640	0.365	0.9452	1.8–2.4
	HS	2.16	1.93	2.13	2.37					
Phosphorus (mg/dL)	TN	4.2	4.66	5.01	3.68	0.42	0.4712	0.2972	0.4471	2.6–6.2
	HS	3.99	4.12	4.13	4.11					
Calcium (mg/dL)	TN	10.41	10.37	10.64	10.08	0.34	0.2462	0.7912	0.7912	9–12
	**HS**	10.23	9.84	10.1	10.01					

SEM, standard error of the means; G, Group; T, Time.

^a^Kaneko (42).

Different letters on the same parameter of the same group show statistical differences: A, B, C = *P* < 0.01. Different letters at the same time for the same parameter show statistical differences: X, Y = *P* < 0.01.

**Table 6:** Thiobarbituric acid reactive substances (TBARs), hydroperoxides, protein carbonyls, ferric reducing antioxidant power (FRAP), superoxide dismutase (SOD), catalase (CAT), and glutathione peroxidase (GSPx) serum concentration in dogs in thermoneutrality (TN) and heat stress (HS) condition for 90 days.

**Parameter**	**Group**	**Time (days)**				**SEM**	***p*** **-value**		
		**0**	**30**	**60**	**90**		**G**	**T**	**G** × **T**
TBARs (mmol/mL)	TN	1.24	1.21^X^	1.24 ^X^	1.12^X^	0.10	< 0.0001	< 0.0001	< 0.0001
	HS	1.49^A^	2.47^B, Y^	2.86 ^B, Y^	3.03^B, Y^				
Hydroperoxides (mmol/mL)	TN	5.24	5.33	5.81	4.59	0.49	0.5542	0.3071	0.8362
	HS	4.99	5.39	5.82	5.37				
Protein carbonyls (μmol/mg Protein)	TN	98.87	91.2	92.14	93.43	2.63	0.0501	0.1922	0.6671
	HS	95.86	96.62	95.88	98.86				
FRAP (μmol TE/mL)	TN	48.36	46.86	46.71	46.43	0.38	< 0.0001	< 0.0001	< 0.0001
	HS	49.32	47.95	49.67	48.7				
SOD (U/mL)	TN	48.41	49.84^X^	54.52^X^	54.07^X^	2.14	< 0.0001	< 0.0001	< 0.0001
	HS	45.82^A^	30.05^B, Y^	33.24^B, Y^	34.98^B, Y^				
CAT (U/mL)	TN	2.88	3.21	3.33^X^	3.28^X^	0.12	< 0.0001	< 0.0001	< 0.0001
	HS	2.12^A^	2.43^A^	1.53^B, Y^	1.62^B, Y^				
GSPx (nmol NADPH ox/mL)	TN	2.32	2.40	2.42^x^	2.76^x^	0.03	0.0464	0.0312	0.0222
	HS	2.43^a^	2.24^a^	1.71^b, y^	1.81^b, y^				
Cortisol (μmol/L)	TN	0.02	0.04^X^	0.03^X^	0.03^X^	0.41	< 0.001	< 0.001	< 0.001
	HS	0.03^A^	0.88^B, Y^	1.89^C, Y^	1.98^C, Y^				
BAP	TN	2138.78	2174.44	2246.67^x^	2281.11^X^	159.33	0.0027	< .0001	< .0001
	HS	2211.11^Aa^	2057.73^Aa^	1719.70^b, y^	1445.99^B, Y^				
ROS	TN	70.22	70.44	71.56	75.88^X^	11.93	0.0012	< 0.0001	< 0.0001
	HS	67.44^A^	79.44^A^	100.67^A^	155.00^B, Y^				

SEM, standard error of the means; G, Group; T, Time.

Different letters on the same line show statistical differences during time in the same group: A, B, = *P* < 0.01; a, b = *P* < 0.05. Different letters on the same row show statistical differences between groups at the same time: X, Y = *P* < 0.01; x, y = *P* < 0.05.

**Table 7:** Volume, concentration, total sperm number (TSN), velocity average pathway (VAP), straight line velocity (VLS), curvilinear velocity (VCL), amplitude of lateral head displacement (ALH), beat-cross frequency (BCF), straightness (STR), and linearity (LIN) of second fraction of ejaculate in dog in thermoneutrality (TN) and heat stress (HS) condition at 0, 30, 60 and 90 days after the starting of the experimentation.

**Parameter**	**Condition**	**Time (days)**							
		**0**	**30**	**60**	**90**	**SEM**	**G**	**T**	**G** × **T**
Volume (mL)	TN	4.86	3.65	3.60	4.25	0.75	0.0363	0.6834	0.7717
	HS	3.25	3.08	2.96	2.41				
Concentration (M/mL)	TN	268.33	254.50^X^	308.00^X^	320.16^X^	28.00	< 0.0001	0.8701	0.4286
	HS	262.86^A^	139.58^BY^	147.22 ^BY^	144.84 ^BY^				
TSN (M)	TN	1,247.25	995.35^x^	1,045.25^x^	1,285.44^x^	421.52	0.0385	0.0124	0.0289
	HS	932.71^a^	436.75^by^	427.65^by^	361.75^by^				
VAP (μm/s)	TN	103.20	108.48^x^	110.10^X^	110.16^X^	5.60	< 0.0001	0.9784	0.6546
	HS	90.50	89.83^y^	86.00^Y^	84.33^Y^				
VSL (μm/s)	TN	92.00	90.16	89.83	84.16	2.60	0.5912	0.7004	0.4113
	HS	88.60	84.74	82.76	84.84				
VCL (μm/s)	TN	179.00^A^	176.33^Ax^	166.00^a^	147.16^Bb^	6.80	0.0017	0.4502	0.0078
	HS	171.98^a^	150.34^by^	153.20^b^	158.06^b^				
ALH (μ)	TN	5.16	5.66	6.00	5.45	0.40	0.4558	0.3289	0.2893
	HS	5.24	5.72	5.70	5.60				
BCF (Hz)	TN	25.16	25.66	23.66	22.83	1.10	0.0609	0.3474	0.0731
	HS	23.50	22.18	23.38	23.20				
STR (VSL/VAP)	TN	79.80	83.60	81.40	83.60^X^	1.50	0.0075	0.1989	0.0054
	HS	82.33^A^	80.83^A^	78.66^a^	74.50^BbY^				
LIN (VSL/VCL)	TN	53.80	54.60	56.40	56.00^x^	1.80	0.6682	0.4452	0.0430
	HS	58.50^A^	55.33	54.16	50.66^By^				

SEM, standard error of the means; G, Group; T, Time.

Different letters on the same line show statistical differences during time in the same group: A, B = *p* < 0.01; a, b = *p* < 0.05; Different letters on the same row show statistical differences between groups at the same aging time: X, Y = *p* < 0.01; x, y = *p* < 0.05.

**Table 8:** Total motility, progressive motility, rapid, medium, slow, and static movements of spermatozoa in dog in thermoneutrality (TN) and heat stress (HS) condition for at 0, 30, 60 and 90 days after the starting of the experimentation.

**Parameter**	**Condition**	**Time (days)**							
		**0**	**30**	**60**	**90**	**SEM**	**G**	**T**	**G** × **T**
Total motility (%)	TN	83.80	87.22	89.70^X^	88.26^X^	3.30	0.0100	0.3400	0.0325
	HS	89.33^A^	88.33^A^	81.00^Y^	75.66^BY^				
Progressive motility (%)	TN	74.80	70.46	72.94^X^	72.68^X^	2.50	0.0721	0.2312	0.0005
	HS	69.50^Aa^	68.00^Aa^	61.50^bY^	55.16^BY^				
Rapid (%)	TN	90.60	83.20^X^	86.00^X^	84.60^X^	3.50	< 0.0001	0.6462	0.0366
	HS	90.83^Aa^	74.00^bY^	71.83^bY^	66.33^BY^				
Medium (%)	TN	10.00	10.20	10.40	10.60	1.58	0.2275	0.4146	0.5126
	HS	9.16	10.00	9.83	9.50				
Slow (%)	TN	6.20	5.00^X^	6.20^X^	6.00 ^X^	1.70	< 0.0001	0.0630	0.0359
	HS	5.16^A^	16.83^BY^	18.50^BY^	17.16^BY^				
Static (%)	TN	3.20	1.20^x^	1.40^x^	1.60^x^	1.60	< 0.0001	0.9106	0.2788
	HS	2.00	3.66^y^	5.66^y^	5.16^y^				

SEM, standard error of the means; G, Group; T, Time.

Different letters on the same line show statistical differences during time in the same group: A, B = *p* < 0.01; a, b = *p* < 0.05; Different letters on the same row show statistical differences between groups at the same aging time: X, Y = *p* < 0.01; x, y = *p* < 0.05.

### 3.1 Discussion

The aim of this study was to evaluate the effects of high environmental temperatures on the clinical and biochemical parameters, oxidative status, and sperm parameters of dogs. It is well-known that thermal stress affects the biological functioning of various organs, resulting in a decrease in their overall performance (41). In this study, the data obtained from the serum biochemical and electrolyte profiles showed that some parameters were affected by heat stress, although they never showed values over the physiological range of the species (42, 43). As regarding serum biochemical profile, total protein, albumin, glycemia, ALT, AST and ALP were affected by heat stress. In particular, total protein, albumin, urea and glycemia reduced in HS dogs, while ALT, AST, and ALP increased after heat exposition. These variations are due to the direct impact of high temperatures on the body's thermal regulation process. The decrease of total protein and albumin could be due to thermal injury that cause cellular necrosis through protein denaturation (44). Following peripheric vasodilation, there is a constriction of capillaries in internal organs like the liver. This constriction can lead to liver cell damage, reflected in increased concentrations of AST and ALT in animals exposed to thermal stress (45). Moreover, heat stress induced hypoglycemia, as just reported by other authors, due to higher metabolism. It increased dilatation of blood vessels and consequently increased absorption of insulin (46, 47). In addition to all this, chloride levels and urea levels have also changed, showing a decrease under heat stress conditions. Dogs in such circum-stances tend to drink more, leading to overhydration and hence these results (48). An increase in cortisol levels was noticed as a reaction to heat stress (49). Circulating cortisol levels have been recognized as a highly sensitive indicator of heat stress due to decreased tolerance to extreme climates. Dogs experiencing heat stress have been observed to exhibit cortisol levels exceeding the normal range (50). The increasing values of cortisol observed in this study indicate that dogs were experiencing the heat stress effects (46). It is known that heat also induces oxidative stress (8, 51). Also in this study, it was demonstrated that the parameters of oxidative stress (TBARs and ROS) were increased in HS groups, while antioxidants (SOD, CAT, GSPx, and BAP) were reduced. This is indicative of oxidative stress, that is the unbalance between the production of oxidation by-products and reduction of antioxidant enzymatic activity (52). Currently, there are no studies that can substantiate such evidence because, based on our current knowledge, no researcher has yet studied the oxidative stress during heat stress in dogs. Considering the damage caused by testicular heat stress, it is important to investigate the alterations caused by heat stress in sperm development to improve animal welfare in dogs living outdoors in the summer months. In other species (bovines, rabbits, mice), instead, it was demonstrated how the heat stress induces oxidative stress and interferes with reproduction (53–55). In livestock industry, global warming has negatively influenced animal production performance, in addition to their wellbeing and welfare. In the present study, heat induced-oxidative stress affected the sperm parameters. Prior to heat stress (T0), every parameter examined in both group of dogs (volume, motility, vigor, sperm concentration, total sperm count, and morphology) fell within the range deemed as normal (21, 24). In the summer months, with an increase in THI, the sperm parameters worsen. It was demonstrated that ROS, produced after scrotal exposure to ambient high temperatures, can stop spermatogenesis (22, 56). The decrease in sperm concentration, velocity average pathway, straight line velocity, curvilinear velocity, amplitude of lateral head displacement, straightness, and linearity is a result of heat stress-induced generation of reactive oxygen species, which causes cellular damage and the start of apoptosis (17, 57). Apoptosis may result in sperm cells death when heat stress acts on the testes (58). This study has highlighted how heat stress can alter the oxidative status of male dogs, promoting increased production of oxidative catabolites and reducing antioxidant enzyme activity. This is also observed at the spermatic level with an increased production of ROS. In fact, the current study demonstrates the detrimental effect of high environmental temperatures on sperm quality in dog. This occurs due to the direct effect of heat on spermatozoa and the production of ROS which damage sperm cells.

The negative impact of a heat stress on quantitative and qualitative sperm traits has been demonstrated in a variety of species (59, 60). In bulls, a notable decrease in sperm motility (MOT), velocity (VEL), viability (VIA), acrosome integrity (ACR), and progressive motility index (PMI) has been documented during summer, accompanied by an increase in sperm abnormalities, particularly in the head, midpiece, and tail regions (61). Similarly, a morphology analysis conducted on Bos taurus and Bos indicus exposed to an environmental temperature of 40°C revealed an increase in misshapen sperm heads (especially pyriform heads), decapitated spermatozoa, and bent tails (62). Likewise, cats exhibited the poorest seminal quality on high-temperature days (63). The negative impact of heat stress on sperm quality has also been reported in rams (64), boars (65), and men (66). Consistent with these findings, we observed a detrimental effect of heat stress on sperm motility, velocity, sperm morphology, and viability in dogs. To the best of our knowledge, this is the first report demonstrating the effective-ness of heat stress in assessing the degree of stress induced by climatic conditions in dogs. Although extensively studied in farm animals to mitigate economic losses, research on this topic has been limited in wild animals and pets. In wild animals, studies have focused on ensuring the wellbeing and welfare of captive animals, addressing behaviors, habitat enrichment, or stressors. However, little is known about heat stress and temperature thresholds for many wild species (67). There exists a temperature threshold, influenced by the duration of exposure, beyond which germ cell degeneration occurs, as demonstrated in mice and dogs (17). There are several factors that influence seminal testosterone and semen production in dogs, for example the characteristics of the subject, the length of the day, the environmental humidity (68). All these factors were standardized in the two groups, to show the effect of temperature alone in the selected dogs. This study has limitations: the imperfect standardization of the groups, with physical and sperm production differences in the between them. Furthermore, it was not possible to follow the dogs for some months after the end of the trial to have more data after the summer season.

## 4 Conclusion

This study has highlighted how heat stress can alter the oxidative status of male dogs, leading to increased production of oxidative catabolites and a reduction in antioxidant enzyme activity. This phenomenon is also observed at the spermatic level, with a marked increase in the production of ROS. In fact, the current research demonstrates the detrimental effect of high environmental temperatures on sperm quality in dogs. This occurs due to the direct impact of heat on spermatozoa, coupled with the increased production of ROS, which damages sperm cells. The effect was observed in animals exposed to high temperatures during the summer months in which the study was conducted, leading to a decrease in reproductive performance in male dogs kept outdoors and subjected to extreme heat. It is important to note, however, that one limitation of the study is the lack of complete homogeneity among the groups in terms of breed. While the study provides valuable insights into the impact of heat stress on oxidative status and sperm quality in dogs, further research is required to gain a deeper understanding of these effects.

## Data Availability

The raw data supporting the conclusions of this article will be made available by the authors, without undue reservation.
